# Malaria and other vector-borne infection surveillance in the U.S. Department of Defense Armed Forces Health Surveillance Center-Global Emerging Infections Surveillance program: review of 2009 accomplishments

**DOI:** 10.1186/1471-2458-11-S2-S9

**Published:** 2011-03-04

**Authors:** Mark M Fukuda, Terry A Klein, Tadeusz Kochel, Talia M Quandelacy, Bryan L Smith, Jeff Villinski, Delia Bethell, Stuart Tyner, Youry Se, Chanthap Lon, David Saunders, Jacob Johnson, Eric Wagar, Douglas Walsh, Matthew Kasper, Jose L Sanchez, Clara J Witt, Qin Cheng, Norman Waters, Sanjaya K Shrestha, Julie A Pavlin, Andres G Lescano, Paul CF Graf, Jason H Richardson, Salomon Durand, William O Rogers, David L Blazes, Kevin L Russell, the AFHSC-GEIS Malaria and Vector Borne Infections Writing Group

**Affiliations:** 1 Armed Forces Health Surveillance Center, 2900 Linden Lane, Silver Spring, MD 20910, USA; 2 Force Health Protection and Preventive Medicine, 65th Medical Brigade, Unit 15281, APO AP 96205-5281 USA (Republic of Korea; 3 US Naval Medical Research Center Detachment (NMRCD), Centro Medico Naval “CMST,” Av. Venezuela CDRA 36, Callao 2, Lima, Peru; 4 US Army Medical Component Armed Forces Research Institute of the Medical Sciences, APO AP 96546, Bangkok, Thailand; 5 US Army Medical Research Unit Kenya, United States Embassy, ATTN: MRU, United Nations Avenue, Post Office Box 606, Village Market, 00621 Nairobi, Kenya; 6 US Naval Medical Research Unit Number 3, Extension of Ramses Street, Adjacent to Abbassia Fever Hospital, Postal Code 11517, Cairo, Egypt; 7 US Navy Medical Research Unit-2, U.S. Embassy Unit 8166 Box P, APO AP 96546, Phnom Penh, Cambodia; 8 Australian Army Malaria Institute, Weary Dunlop Drive, Gallipoli Barracks, Enoggera, QLD 4051 Australia; 9 Naval Medical Research Center, 503 Robert Grant Ave. Silver Spring, MD 20910, USA

## Abstract

Vector-borne infections (VBI) are defined as infectious diseases transmitted by the bite or mechanical transfer of arthropod vectors. They constitute a significant proportion of the global infectious disease burden. United States (U.S.) Department of Defense (DoD) personnel are especially vulnerable to VBIs due to occupational contact with arthropod vectors, immunological naiveté to previously unencountered pathogens, and limited diagnostic and treatment options available in the austere and unstable environments sometimes associated with military operations. In addition to the risk uniquely encountered by military populations, other factors have driven the worldwide emergence of VBIs. Unprecedented levels of global travel, tourism and trade, and blurred lines of demarcation between zoonotic VBI reservoirs and human populations increase vector exposure. Urban growth in previously undeveloped regions and perturbations in global weather patterns also contribute to the rise of VBIs. The Armed Forces Health Surveillance Center-Global Emerging Infections Surveillance and Response System (AFHSC-GEIS) and its partners at DoD overseas laboratories form a network to better characterize the nature, emergence and growth of VBIs globally. In 2009 the network tested 19,730 specimens from 25 sites for *Plasmodium* species and malaria drug resistance phenotypes and nearly another 10,000 samples to determine the etiologies of non-*Plasmodium* species VBIs from regions spanning from Oceania to Africa, South America, and northeast, south and Southeast Asia. This review describes recent VBI-related epidemiological studies conducted by AFHSC-GEIS partner laboratories within the OCONUS DoD laboratory network emphasizing their impact on human populations.

## Introduction

Vector borne infections (VBIs) such as malaria, dengue fever, yellow fever, scrub typhus, and plague comprise a significant proportion of the global infectious disease burden. These diseases account for more than half of the priority diseases in the Special Program for Research and Training in Tropical Diseases, a scientific collaboration of the World Health Organization (WHO), the United Nations Children's Fund (UNICEF), the United Nations Development Program and the World Bank. High priority VBIs include malaria, lymphatic filariasis, leishmaniasis, African trypanosomiasis, onchocerciasis, dengue and Chagas disease, many of which are also considered to be neglected diseases.

Typically defined as infections transmitted from the bite or mechanical transfer of an arthropod vector [1], VBIs are of growing importance in an era of global travel. Increasingly blurred lines of demarcation between human and zoonotic disease reservoirs and the unpredictable effect of climate change on the distribution and behavior of arthropod vectors also contribute to the rise of VBIs. Disproportionately high VBI burdens fall in areas with poor health infrastructure, underscoring the need to develop and maintain responsive surveillance systems capable of detecting VBI outbreaks in resource-constrained environments.

The Defense Department has long operated a network of medical research laboratories principally to conduct research and development on diseases of military impact. These laboratories commonly referred to as DoD OCONUS laboratories, have emphasized VBIs in their infectious disease research portfolios. The historical rationale for this emphasis on VBIs is the need to conserve and maintain the health and capacity of troops while operating in a variety of settings with increased exposure to disease-carrying arthropod vectors. The potential impact of vector-borne disease on military populations is illustrated by General Douglas MacArthur, who, referring to the impact of VBI on World War II forces, famously lamented, “This will be a long war, if for every division I have facing the enemy, I must count on a second division in the hospital with malaria, and a third division convalescing from this debilitating disease.” [2].

The creation of DoD-GEIS was inspired by the 1992 Institute of Medicine (IOM) report on emerging infections and formally tasked through the June 1996 Presidential Decision Directive (NSTC-7) on emerging infections, which expanded on the original IOM report. The merging of DoD-GEIS with the DoD OCONUS laboratory research mission leveraged the laboratories’ strengths—capacity for high-quality hypothesis-driven scientific rigor and established host-nation relationships—and sought to incorporate the additional components of global surveillance, training and response to infectious disease threats. In addition, existing OCONUS laboratory programs oriented toward the development of new vaccines, drugs and diagnostics for diseases in the developing world would benefit from the acquisition of timely surveillance data to help guide their efforts.

The inception and subsequent execution of the program coincides with a period when DoD’s public health concerns, expressed by General MacArthur decades ago, increasingly converged with those of the developing world. Since the end of the Cold War, DoD personnel increasingly have been deployed to to render humanitarian assistance in regions of political and economic turmoil and natural disasters. The 2010 Haiti earthquake relief effort, during which several DoD personnel contracted *Plasmodium falciparum* malaria [3], exemplifies this risk.

The AFHSC-GEIS network and its OCONUS laboratory partners conduct VBI surveillance of in arthropod vectors, animals and humans, with emphasis on surveillance of vector-borne diseases in humans, the primary host of interest. The VBI program aims to characterize present occurrences of VBI in humans as well as support the AFHSC-GEIS predictive surveillance program [4] by validating the capability of satellite remote sensing, ecological niche modeling, and arthropod vector and zoonotic reservoir surveillance to predict the risk of VBI transmission to humans. This report reviews the 2009 accomplishments of the AFHSC-GEIS and DoD OCONUS laboratory VBI surveillance network.

## Malaria surveillance

One of the world’s largest malaria drug resistance surveillance networks is maintained by AFHSC-GEIS, with sites in Africa, South America, the western Pacific islands, and northeast, south and Southeast Asia. The emphasis on malaria drug resistance is well justified. Since World War II, an inexorable pattern of resistance has rendered once-useful malaria treatments, such as chloroquine, sulfadoxine/pyrimethamine and mefloquine, ineffective in large parts of the malaria-endemic world. Despite tremendous progress, there is still no vaccine that prevents infection or disease. This underscores the threat posed by emerging drug resistance and the importance of effective surveillance systems to detect the onset of resistance and assure optimal treatments.

In 2009, the AFHSC-GEIS laboratory network analyzed 19,730 specimens from 25 sites spanning malaria-endemic regions using techniques, such as molecular characterization of resistance genes and *in vitro* drug sensitivity assays to determine inhibitory concentrations against a battery of common malaria drugs. Some sites are also capable of conducting therapeutic efficacy and complex pharmacokinetic *in vivo* studies to better understand drug-parasite interactions.

Today, the most effective anti-malarial drugs are those in the artemisinin class. The artemisinins, derived from *Artemisia annua*, have been used in Chinese medicine for centuries under the name Qinghaosu and eventually incorporated as first-line treatment for *P. falciparum* worldwide. Compared to other malaria drugs such as mefloquine and chloroquine, artemisinin derivatives are vastly superior in terms of safety, tolerability and efficacy, and—until recently—unmarred by resistance. Although the artemisinins have been used on their own as a single-agent therapy, fears over the possible development of resistance have given rise to the concept that malaria treatments should follow the examples adopted for tuberculosis and human immunodeficiency virus (HIV)—that combining drugs with differing mechanisms of action will both optimize patient outcomes and minimize the risk that resistance will develop. Such artemisinin combination therapies (ACTs) contain a short-acting artemisinin component to quickly reduce parasite burden and clinical symptoms and a longer-acting partner drug to clear remaining parasites. This approach is now recommended by WHO as the first-line treatment for uncomplicated *P. falciparum* malaria in all endemic countries.

The major global investment in ACTs has been threatened recently by increasing ACT treatment failures on the Thailand-Cambodia border, an area historically considered an epicenter of drug-resistant malaria. Between 2002 and 2004, increased parasite clearance times and unusually high failure rates with the ACT regimens artesunate-mefloquine and artemether-lumefantrine were being reported on both sides of the border [5,6] and begged the question of whether resistance to the artemisinin component or its partner drug was the culprit. To further explore this question, the Armed Forces Research Institute of Medical Sciences (AFRIMS) in Bangkok, Thailand, conducted a combined *in vivo/in vitro* artemisinin-monotherapy efficacy study to isolate the specific effect that artemisinin resistance may have played in the ACT failures. Although the 28-day cure rate of the artemisinin monotherapy regimen was greater than 90 percent, two subjects failed artemisinin therapy despite the documentation of adequate plasma drug levels, direct observation of medication doses and up to a 4.3-fold higher IC_50_ concentration than the overall mean. This report, the first to document clinical artemisinin resistance, raised serious concerns that resistance to the artemisinin component of ACTs had developed [7].

In 2009, AFRIMS continued to expand its drug-resistance efforts to examine possible countermeasures to combat the potential spread of resistant strains. No previous investigations had been conducted to determine the optimal artemisinin dose regimens that most effectively clear *P. falciparum* parasites. While current dosing regimens in widespread use were effective for relatively sensitive parasites, the specter of artemisinin resistance raised the question of whether higher doses might more effectively clear resistant strains.

AFRIMS conducted a study exploring dose-effect relationships with particular attention to the safety and tolerability limitations imposed at doses higher than previously tested in humans. Subjects with uncomplicated *P. falciparum* malaria were given a total of 14, 28 or 42 mg/kg of oral artesunate divided over a week and followed for clinical and parasitological endpoints. Safety and efficacy findings of the highest dose were particularly critical since demonstration of improved cure rates at higher doses might be critical in designing higher-dose regimens for widespread deployment. Unfortunately, enhanced efficacy was not noted at the high dose arm, either in parasite clearance times or cure rates. Furthermore, transient neutropenia was observed at the 42-mg/kg dose, suggesting toxicity to myeloid progenitor cells. Together, these safety and efficacy findings allowed AFRIMS to characterize an important dose-limiting toxicity of the artemisinins—a valuable data set in light of worldwide deployment of ACTs [8].

To further characterize the artemisinin resistance phenomenon in 2009, Naval Medical Research Unit Number 2 (NAMRU-2) completed a *P. falciparum* therapeutic efficacy study in the Chumkiri District of Kompot Province, approximately 100 km south-southwest of Phnom Penh, Cambodia. In contrast to the areas along the Thai-Cambodian border, where AFRIMS had conducted its drug resistance studies, the Chumkiri site was chosen in part to determine whether ACT resistance had spread to points farther east. The NAMRU-2 study documented an 18.8 percent treatment failure rate employing the national standard regimen of artesunate-mefloquine combination therapy. Treatment failure was associated with increased *pfmdr1* copy number, higher initial parasitemia, higher mefloquine IC_50_ and longer parasite clearance times [9]. This study demonstrated that resistance to the artesunate-mefloquien regimen extended beyond the highly suspected areas of drug resistance along the Thailand-Cambodia border, highlighting the need to expand containment efforts to include Kampot Province.

The increased failure rates of ACTs and the confirmation of resistance to the artemisinin class documented by AFRIMS and NAMRU-2 were regarded as a regional and global emergency. If ACT treatment failures along the Thai-Cambodian border follow the historical precedent of chloroquine (CQ) and sulphadoxine/pyrimethamine (SP) resistance, ACT regimens could be compromised globally within a few years, leading to the frightening possibility of compromising effective treatments for the enormous biomass of *P. falciparum* parasites in sub-Saharan Africa at immense cost to human life. In response, the international malaria community is mounting an aggressive campaign to contain these resistant parasites [10].

This concern is well founded. Specifically, the phenomena of CQ and S/P resistant *P. falciparum* are well described in Africa and been demonstrated to have spread from Southeast Asia to Africa in so-called “selective sweeps” [11,12], suggesting that history may repeat itself with the newly discovered artemisinin resistance phenomenon in Southeast Asia. The Malaria Drug Resistance (MDR) laboratory of the U.S. Army Medical Research Unit – Kenya (USAMRU-K), based in Kisumu, in collaboration with the Kenya Medical Research Institute and Kenya Ministry of Health, has monitored in vitro malaria drug sensitivity and molecular marker profiles across Kenya since 1995. In 2009, *P. falciparum* drug resistance surveillance efforts focused in western Kenya at three district hospitals in Kisumu, Kericho, and Kisii. A total of 213 *P. falciparum* specimens were collected and frozen for later drug susceptibility profiling against a panel of six to twelve antimalarial drugs. Concurrently, 182 samples were examined for molecular markers associated with *P. falciparum* drug resistance, including Pfmdr1 copy number and select point mutations in Pfmdr1, Pfcrt, and PfATPase6. Data analysis indicates that decreasing but high levels of chloroquine-resistant, low levels of mefloquine-resistant, and no artemisinin-resistant parasite profiles were present among samples assessed. Reassuringly, i*n vitro* drug sensitivity patterns and mutation rates suggest that overall *P. falciparum* drug resistance was stable in Kenya from 2006 through 2009.

Future USAMRU-K drug resistance surveillance efforts will emphasize monitoring artemisinin susceptibility of Kenyan isolates with an integrated approach to correlate *in vitro* drug sensitivity testing with clinical *in vivo* resistance assessments. Artemisinin resistance monitoring is particularly timely in light of the recent adoption of the ACT artemether-lumefantrine as first-line therapy for uncomplicated *P. falciparum*[*13*]. Timing is optimal to now establish baseline laboratory and clinical resistance data against which future assessments can be compared, both within Kenya and globally.

Malaria surveillance efforts at the Australian Army Malaria Institute (AMI) have focused primarily on the prevalence of malaria infection and incidence of drug resistance within the western Pacific region. Efforts in 2009 concentrated on the Vanuatu and the Solomon Islands. In collaboration with the Pacific Malaria Initiative Support Centre, village-based mass blood surveys were conducted and malaria-positive samples were genotyped to determine the prevalence of chloroquine drug-resistance polymorphism within the Pfcrt gene. Twelve codons of Pfcrt were evaluated and a consensus polymorphic profile was established that allowed for comparison between different countries. The establishment of a consensus “genetic fingerprint” of Pfcrt polymorphisms and its correlation with microsatellite markers, not under immune or drug selection, has provided information on the flow of malaria drug resistance genotypes throughout the region. Current data demonstrate the existence of a common consensus genotype in Papua New Guinea, Vanuatu and the Solomon Islands—distinct from genotypes arising in Indonesia and the Philippines. These findings have implications for the manner in which drug-resistant alleles originating in Southeast Asia may spread throughout the western Pacific.

The AFHSC-GEIS malaria program has also been active in surveillance of *Plasmodium vivax* malaria throughout Asia and South America. As opposed to *P. falciparum*, *P. vivax* treatments are complicated by the need to eradicate dormant hypnozoites. Because of the difficulty in assessing which patients harbor undetectable hypnozoites, it is critical to ensure that radical curative regimens employing chloroquine (CQ) and primaquine (PQ) are optimized. Although highly curative when administered for a two-week course, most malaria control programs acknowledge the real-world effectiveness of PQ for hypnozoite eradication to be inadequate, largely due to insufficient compliance with the protracted regimen. Concerns about G6PD-related hemolysis also limit the incorporation of PQ into national treatment guidelines.

In 2009, malaria scientists at the Naval Medical Research Center Detachment (NMRCD) in Peru reported the results of an efficacy study of abbreviated PQ regimens for the prevention *P.vivax* relapses. Patients were treated under direct supervision in three health centers in Iquitos, the largest city of the Peruvian Amazon Basin, with CQ, 25 mg/kg over three days, plus primaquine, in one of three different randomly-assigned regimens: 0.25 mg/kg daily for 14 days, 0.5 mg/kg daily for seven days or 0.5 mg/kg for five days. The regimens selected represent the WHO-recommended regimen, the Pan American Health Organization (PAHO)-recommended regimen, and a shorter version of the PAHO regimen, respectively. Of the evaluable 491 patients, 48 presented with reappearance of parasitemia due to the same vivax genotype after 35 days of initiating the therapy: 27 (16.0 percent) in the five-day arm, 9 (5.8 percent) in the seven-day arm and 12 (7.4 percent) in the 14-day arm (unpublished data). NMRCD concluded that the seven- and 14-day PQ regimens are similar to each other in efficacy and superior to the shorter five-day regimen in preventing relapse of *P.vivax* malaria [14]. Despite the lower efficacy of the five-day regimen, it likely offers some benefit over regimens without a primaquine component.

Although resistance to malaria chemotherapy has traditionally been more problematic for *P. falciparum*, it is also necessary to remain vigilant for resistant *P.vivax* strains. Chloroquine is nearly universally used as the first-line therapy for *P. vivax* malaria due to its high efficacy and low cost, and PQ remains the only drug capable of eradicating hypnozoites from the relapsing *Plasmodia* species *vivax* and *ovale*. As part of the above-mentioned treatment efficacy study, NMRCD investigators uncovered new evidence of the transmission of CQ-resistant *P.vivax* in the Peruvian Amazon Basin. Of the four patients with reappearance of parasitemia, one was determined to be probably resistant to chloroquine when a whole-blood CQ level at the time of reappearance of parasitemia was measured to be 95 ng/mL and pvmdr1 gene sequencing and neutral microsatellite markers analysis revealed the same *P. vivax* genotype in the reappearing and original parasites [15].

In the Republic of Korea, similar concerns that CQ-resistant *P.vivax* may have caused an increased caseload from 2005 to 2007 led GEIS partners to conduct a prophylactic efficacy study in 2009. Enrolled into the study were 142 *vivax* malaria patients, most of whom were participants in the Korean Army hydroxychloroquine (HCQ) chemoprophylaxis program. To rule out non-compliance as a cause for prophylaxis failure, plasma HCQ metabolite levels were determined on the day of enrollment. Most soldiers with “breakthrough” *vivax* malaria infections harbored undetectable HCQ levels. Fourteen of 127 (11 percent) of subjects were determined to have HCQ levels >100 ng/mL, meeting established criteria for biological resistance or suspected biological resistance.

The study was the first to describe chloroquine-resistant *P.vivax* prophylaxis failures on the peninsula and raises concerns given the widespread use of HCQ and chloroquine for both treatment and prophylaxis of *vivax* malaria in Korea. Although noncompliance may have contributed to the increased caseload, the widespread use of unmonitored HCQ prophylaxis raised concerns that chloroquine resistance, a phenomenon previously undocumented in Korea, may have contributed [16].

## Surveillance of other VBIs in human populations

Although malaria shares common clinical features with other VBIs, the difficulty encountered by clinicians in rendering accurate diagnoses of other VBIs is hampered by the fact that they are not easily diagnosed at the pathogen level. Many studies have corroborated the need for laboratory-based diagnostics in order to distinguish one etiologic cause of undifferentiated fever from another. This is particularly applicable to VBIs, which generally present as undifferentiated fever [17-19].

Throughout the AFHSC-GEIS laboratory network, efforts were made in 2009 to enhance or establish hospital-based febrile illness surveillance platforms in Azerbaijan, Bolivia, Cambodia, Ecuador, Georgia, Kenya, Nepal, Paraguay and Peru in an effort to guide clinical treatments for undifferentiated febrile illness—many of which were caused by VBIs. Working in collaboration with local Ministries of Health and other institutions, such as the U.S. Army Medical Research Institute of Infectious Diseases, Walter Reed Army Institute of Research, and the U.S. Centers for Disease Control and Prevention, specimens collected from patients with acute febrile illness at hospitals and clinics have allowed AFRIMS, NMRCD, NAMRU-2, Naval Medical Research Unit Number 3 (NAMRU-3), and U.S. Army Research Unit-Kenya (USAMRU-K) laboratories to conduct etiological agent identification, monitor the prevalence of VBIs within the AFHSC-GEIS network regions, and better understand VBI epidemiology and geographic distribution.

In 2009, AFHSC-GEIS partner laboratories conducted etiological agent identification of non-malaria VBIs on more than 10,000 specimens from 43 sites in nine countries. The use of techniques such as viral culture, polymerase chain reaction (PCR) assays and enzyme-linked immunosorbent assays (ELISA) led to the discovery of new pathogens and serotypes new to regional areas. For instance, Guaroa virus (GROV) infection, transmitted by *Anopheles neivai*, was documented first in Colombia, and later in Brazil and Panama. Through the febrile illness surveillance study by NMRCD researchers [20], several GROV cases were documented in febrile illness patients from Bolivia and Peru by ELISA detection of IgM-specific antibodies. Prior to this study, GROV infection had not been documented in Bolivia and Peru but should now be considered in the differential diagnosis for febrile illness of unknown etiology in that region.

Recent reports of vector-borne diseases circulating in Nepal have described the first documented case of dengue virus [21] and the spread of all dengue virus serotypes circulating in 2008 [22]. To further expand on these findings, AFRIMS initiated a hospital-based study to determine etiologies of undifferentiated febrile illnesses at four hospitals in Nepal (two in Kathmandu, one in Pokhara and one in Bharatpur). Through fiscal year 2009, 163 patients had been enrolled.

Although testing is still in progress, laboratory testing has confirmed or suggested a diagnosis in over 75 percent of cases enrolled to date. In addition to non-VBIs detected (*Pseudomonas aeruginosa* (one), *E. coli* (one), *Salmonella typhi* (three), *Salmonella paratyphi* A (four), leptospirosis (37), hepatitis A virus (four), hepatitis C virus (one), brucellosis (16), influenza A H3 (six), influenza A H1Sw (two), influenza B (two) in the initially enrolled patients, 28.2 percent had a VBI: scrub typhus (19), murine typhus (three), Japanese encephalitis (JE) (two), primary dengue infection (12), secondary dengue infection (nine), and malaria (non-*falciparum*) (one). Diagnostic analyses yet to be completed include testing for *Bartonella* infection, which has not previously been determined to be a cause of illness in Nepal, and pathogen discovery for unrecognized viruses. The study continues enrollment through 2010 to determine the seasonal risk for VBI and other infections in Nepal.

Hospital-based surveillance conducted by the AFHSC-GEIS partners provides practitioners with critical information used to treat patients, and for implementing disease prevention and control measures. An example is provided by a 2009 seroprevalence study conducted by NAMRU-3 researchers in Azerbaijan, which surveyed 68 patients for West Nile virus (WNV), *Rickettsia typhi*, hepatitis A, Q fever, leptospirosis and brucellosis serologies. Of 68 patients screened, 8.8 percent were positive for leptospirosis, 7.3 percent were positive for *Rickettsia typhi* IgG, 93 percent contained anti-hepatitis A virus antibodies, 31 percent showed Q-fever IgG antibodies, and 16 percent were reactive in brucellosis screening. The data obtained from the study provided clinicians with a better understanding of the risks and exposures for regionally relevant infections, in turn supporting improved treatments. Ancillary benefits of the study included the training and laboratory infrastructure enhancements that enabled the Azerbaijan Ministry of Health to better meet public health needs.

Human seroprevalence of hantaviruses, arenaviruses, brucellosis, WNV, Crimean Congo hemorrhagic fever (CCHF), leptospirosis, rickettsias, and several other vector-borne and zoonotic diseases have also been conducted throughout the network. For example, in 2005, Nepal experienced an outbreak of JE, and an outbreak response executed by WHO and the Nepal Ministry of Health collected samples from acute encephalitis patients. Serological testing, however, revealed that only 35 percent were positive for the disease. In 2009, using randomized JE-negative samples, AFRIMS researchers tested acute illness serum for the presence of IgM to dengue virus, Chikungunya virus, WNV, *Leptospira* and *Brucella*. Of 286 samples, AFRIMS found 75 patients (26.2 percent) with antibodies against *Leptospira*, 18 (6.3 percent) with *Brucella* antibodies and five (1.7 percent) with Chikungunya antibodies. No samples were positive for exposure to dengue virus or WNV. Although VBIs made up a small percentage of samples not positive for JE, this was the first time Chikungunya infection had been documented in Nepal.

Not only is characterization of pathogens responsible for febrile diseases of considerable assistance to clinicians rendering care to individual patients, but it also allows for temporal and spatial tracking of disease and pathogen strains. In Cambodia, NAMRU-2 investigators have tested specimens collected from 5,362 patients for dengue virus, rickettsial infections, hantavirus infections and other pathogens, and determined dengue-2 and dengue-4 serotypes as the predominant circulating strains by real-time PCR. Determination of circulating dengue virus serotypes is particularly relevant in light of the fact that heterotypic strain infection might increase clinical disease severity through an antibody-dependent enhancement mechanism [23].

## Surveillance of VBIs in animals and vectors

While not a major focus of the review, vector and animal surveillance can play a role in mitigating the human impact of emerging infections by triggering measures to limit transmission of vector pathogens from vector and animal reservoirs. In 2009, AFHSC-GEIS continued to conduct arthropod surveillance in Afghanistan, Ethiopia, Kenya, Libya, Peru and Thailand in collaboration with host-nation organizations, including Afghani and Libyan National Malaria and Leishmaniasis Control Programs, Universidad Peruano Cayetano Heredia, Combined Joint Task Force-Horn of Africa, Consortium for National Health Research-Kenya, Cairo University, and several other academic and public health agencies.

Efforts by AFRIMS, NAMRU-3, NMRCD, and USAMRU-K focused on continuing entomology studies for vectors such as mosquitoes, ticks, fleas and mites to test for arboviruses, rickettsiae, and *Leishmania* species. For example, USAMRU-K collected 50,718 ticks and 36,464 mosquitoes to screen for CCHF virus and dengue virus, respectively, in five sites of Kenya: Busia, Kahawa, Kakamega, Kisumu and Isiolo. After initial screening by ELISA for CCHF, three of 16 positive samples were confirmed by PCR and sequenced. Pooled tick samples and mosquito samples were also used for cell culture inoculation for CCHF and dengue virus.

In 2009, AFHSC-GEIS-sponsored animal surveillance activities continued to expand in Kenya, Korea, Peru and Thailand. Tissue and sera from animals, such as rodents and cattle, were collected and screened for the presence of zoonotic pathogens, including arenaviruses, brucellosis, anthrax, leptospirosis and hantaviruses using assays such as Rose Bengal test, Microscopic Agglutination Test, hemaglutinin inhibition assay and ELISA.

A good example of the need for coordinated human-animal surveillance is provided by a seroprevalence study conducted by NMRCD researchers in the Peruvian Amazon region to characterize the epidemiology of spotted fever group (SFGR) and typhus group rickettsial (TGR) infections among humans and domestic pets. From the 1,195 human sera analyzed for SFGR and TGR using anti-SFGR and anti-TGR antibody ELISAs, 521 (43.6 percent) and 123 (10.3 percent) were positive, respectively [9]. Among the 71 canines surveyed for SFGR and TGR, 42 (59.2 percent) were positive for SFGR antibodies and two (2.8 percent) were positive for TGR antibodies. Using a nested PCR, one active SFGR infection was detected among the canines. Among the 17 felines screened, one (7.7 percent) contained SFGR-specific antibodies while none had TGR antibodies. The study demonstrated that prevalence of these rickettsial infections was high within the study population, providing clinicians with a greater awareness of rickettsial infections as a cause of febrile illness in the Iquitos area and that domestic pet owners may be at higher risk [9].

## Discussion

The 1996 Presidential Decision Directive NSTC-7 formally codified and expanded the role of DoD—already a well established contributor in tropical infectious disease research—to include the additional components of global surveillance, training and response to infectious disease threats as part of the DoD-GEIS program. Infectious disease surveillance data obtained from the DoD OCONUS laboratory network are shared freely and published in the peer-reviewed literature to inform local, regional and global health program managers irrespective of national or geographical affiliation. This mutual benefit between host-nation public health programs and DoD has arisen in part because of the unique, communicable nature of infectious diseases. As VBIs increase globally, the need to optimally manage surveillance efforts for maximum impact becomes even more critical.

**Figure 1:**
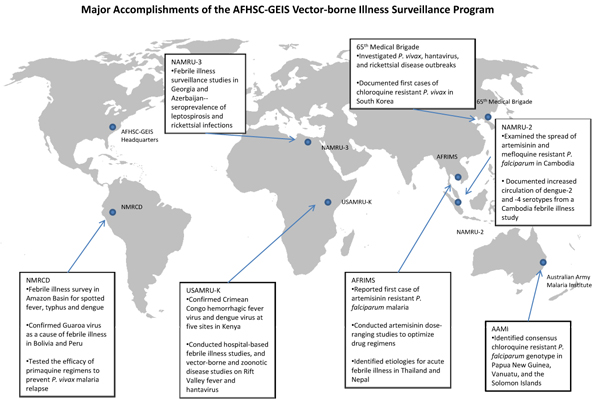
**Map inset:** Major Accomplishments of the AFHSC-GEIS Vector-borne Illness Surveillance Program

The original DoD-GEIS Emerging Infections Prevention Strategy [24] stipulated the need for “standardized sentinel surveillance” to “strengthen and integrate programs to monitor, control and prevent emerging and zoonotic diseases” among its strategic goals, emphasizing drug-resistant malaria, Rift Valley fever, rickettsial infections and dengue fever. In 1998, following the document’s publication, several reviews and panels corroborated the necessity of VBI surveillance [25]. An exhaustive review of global trends in emerging infectious disease attributed 28.8 percent of all emerging infectious disease events to VBIs in the decade following implementation of the original DoD-GEIS Strategy [26]. In 2009, the GEIS VBI surveillance program continued to make significant contributions throughout its global network (Figure 1). However its success, opportunities exist for greater refinement of the program. Enumerated below and in Table 1 are concepts to guide future AFHSC-GEIS VBI surveillance efforts for maximum impact.

**Table 1:** Concepts for the AFHSC-GEIS Vector-Borne Infection (VBI) Surveillance Program

Concept	Rationale
Programmatic organization of surveillance efforts as VBIs is a useful principle to prioritize surveillance efforts	Strict adherence to a VBI-only surveillance program must be balanced with an understanding of prevailing disease threats

VBI surveillance efforts should be closely coordinated with human surveillance	Although disease burden in zoonotic or vector populations is of interest, it is so primarily because of the potential impact on humans

Human VBI case definitions should require laboratory confirmation	Clinical diagnoses of most VBI are of limited sensitivity and specificity; pathogen level identification will guide effective treatment

Case definitions employed should be able to be applied consistently across disparate geographies and longitudinally	If surveillance goal is to map disease in areas of sparse infrastructure, simpler laboratory techniques may be preferable

Advancing technologies should be evaluated continually and incorporated without jeopardizing capability for analysis of longitudinal trend**s**	Balance must be achieved between newer diagnostic technologies and adherence to established laboratory case definitions. Substitution of more sensitive methods may erroneously mimic disease emergence

Standardized laboratory and clinical approaches should be judiciously applied and implemented based on proximal impact on human health	The imperative for a “standardized” surveillance system must be tempered with consideration for the cost-benefit ratio of implementing such standards

To properly power epidemiological studies over a broad geography, laboratory case definitions may diverge from those typically used to guide clinical diagnoses	Example: Studies intended to define the geospatial extent of antimicrobial drug resistance might seek to characterize genotypes from extracted DNA rather than rely on determination of minimum inhibitory concentrations (MICs) from viable organisms, despite the fact that the latter may be more predictive of clinical outcome

Patient samples and associated clinical data must be recorded and maintained in a data and specimen repository in a consistent manner over time	Longitudinal trends can be better assessed if specimens and associated demographic data are catalogued in a manner to allow for retrospective trend analysis

Full use must be made of existing, appropriately collected and catalogued sample sets for retroactive analysis	As technology advances, capability to diagnose previously “undiscovered” pathogens can be applied retroactively to banked specimens to better determine the pace of emergence

## Utility of organizing surveillance initiatives: VBIs vs. non-VBIs

As a practical matter, managing AFHSC-GEIS surveillance into a cohesive program under the familiar nomenclature of “vector-borne infections” is desirable since scientists, government public health structures and medical research communities tend to be organized and resourced accordingly. However, the necessity of conducting surveillance of undifferentiated febrile illnesses that do not fall into specific AFHSC-GEIS pillars (i.e., respiratory, enteric, sexually transmitted or antimicrobial-resistant) illustrates the inadequacy of constraining surveillance efforts for “undifferentiated fever”—a clinical description befitting many VBIs, but not necessarily inclusive of all militarily relevant diseases. Hence, strict adherence to the necessity for arthropod vector transmission may not represent the best framework for the comprehensive programmatic surveillance of diseases that threaten DoD personnel and other military populations. For example, omitted from this review is a rather substantial body of work conducted by AFHSC-GEIS partners specializing in leptospirosis, a disease that does not require an arthropod vector component for transmission, but is transmitted through human contact with infected animal urine.

A requirement for vector-borne transmission also excludes another body of work by AFHSC-GEIS partners in Korea who conducted an outbreak response investigation of hemorrhagic fever with renal syndrome [27]. The investigation localized transmission sites to be near the demilitarized zone (where most U.S. and Korean soldiers conducted field training) and led to the characterization of three novel hantaviruses [28-30]. Although not technically VBIs, these leptospirosis and hantavirus surveillance efforts were prospectively supported by AFHSC-GEIS because of their perceived relevance and potential impact on military and developing-world populations. The demonstrated value of these efforts speaks to the need to judiciously support non-VBI surveillance as conditions warrant rather than hewing to a dogmatic requirement for vector transmission as a condition for programmatic management.

Within in the U.S. military framework, prioritizing disease surveillance for VBIs is consistent with the Infectious Diseases Investment Decision Evaluation Algorithm (ID-IDEAL) framework previously proposed by DoD infectious disease officials [31]. The framework was developed as an algorithmic model to prioritize infectious diseases posing the most substantial threats to deployed U.S. military forces and to guide research and development. The model takes into account disease severity and likelihood of infection on a spatial and temporal basis and assigns a Global Severity Risk Index (GSRI) for DoD personnel. Of the top 20 infectious disease threats prioritized by GSRI, half are VBIs (malaria, dengue fever, Rift Valley fever, Chikungunya, CCHF, sandfly fever, O’nyong-nyong, Sindbis virus, scrub typhus and leishmaniasis).

More recently, a panel composed of DoD infectious disease experts and policymakers issued a priority ranking of infectious disease threats to the U.S. military [32], listing many of the same VBIs identified by the ID-IDEAL model. Taken together, the expert panel document and ID-IDEAL’s GSRI scores may be considered as doctrinal support for an “infectious disease military impact rank” (Table 2) integrating these two reports to guide future surveillance priorities. Incorporation of this index to guide future AFHSC-GEIS endeavors would emphasize surveillance priorities in terms of health impact rather than by mode of transmission.

**Table 2:** Military Infectious Disease Impact Rank: Top 20 febrile illnesses ranked by order of military significance.

Rank	Disease	Median GRSI Score	Medical Force ICDT Rank	Military Impact Index (GRSI/ICDT Rank)
1	Malaria	4949	1	4949
2	Diarrhea, bacterial	5236	3	1745
3	Dengue	3148	2	1574
4	Norovirus and other viral diarrhea	1964*	7	280
5	Leptospirosis	1745	10	174
6	Chikungunya	2608	16	163
7	Rift Valley Fever	2519	24	104
8	HIV/AIDS	728	14	52
9	Meningococcal meningitis	698	17	41
10	Diarrhea, protozoal	411	11	37
11	Crimean-Congo hemorrhagic fever	430	13	33
12	Leishmaniasis	12	5	24
13	Hepatitis E	402	21	19
14	Hemorrhagic fever with renal syndrome	252	15	16
15	Q fever (*Coxiella burnetti*)	92	6	15
16	Rickettsioses	155	19	8
17	Tick Borne encephalitis	134	23	5
18	Influenza	35	8	4
19	Plague	89	18	4
20	Lassa/other arenaviruses	63	22	2

Index calculated as quotient of Infectious Diseases Investment Decision Evaluation Algorithm (ID-IDEAL) Global Risk Severity Index (GRSI)/Integrated Capabilities Development Team (ICDT) rank for diseases listed in both documents. Adapted from refs 27 and 28.

*Mean of all GSRI scores for diarrheal diseases

^+^*Mean of cutaneous*, *visceral and mucosal leishmaniasis GSRI scores*

## Need for “standardization” and requirement for laboratory confirmation

Capitalizing on the expertise resident throughout the OCONUS laboratory network, AFHSC-GEIS’s VBI surveillance efforts have traditionally emphasized laboratory characterization to the pathogen level. The non-specific clinical presentation of most vector-borne diseases makes pathogen-level diagnosis difficult even in the hands of highly experienced clinicians. This emphasizes the need for highly trained laboratory scientists and technicians to use established diagnostic methods to enable informed individual and community prevention and treatment strategies.

The use of the adjective “emerging” as a prefix to “infectious diseases” implies the necessity of determining the temporal and geospatial distribution of any given candidate infectious disease, and assumes consistent clinical or laboratory case definitions are applied over time or between surveillance locales for such comparisons to be valid. Development and implementation of such standard methods, particularly in the realm of laboratory-based diagnoses, are almost always easier said than done, given their logistical and financial costs. In addition, once any given standard approach is implemented, the pace of technological development almost assuredly outdates it. Thus, the imperative for a standardized surveillance system must be tempered with consideration of the cost-benefit ratio involved in implementing any given standardization effort.

The single greatest factor contributing to the success of AFHSC-GEIS has been the traditionally collaborative and close relationship between DoD OCONUS laboratory scientists, public health personnel and their host-nation counterparts. Together, DoD personnel and host nations work to identify, conceive and execute emerging infectious disease surveillance activities based on regional DoD and local Ministry of Health priorities. While this approach has its merits, the emphasis on regional flexibility has come at some expense to creating a standardized surveillance system. The desire for standardization to enable the generation of geographically and longitudinally generalizable data must be balanced with the need to remain responsive to host-nation capabilities and priorities.

A more appropriate approach calls for judicious application of proscriptive standards to maximize the applicability of the overarching surveillance product. Additionally, if laboratory samples cannot be transported to centralized reference laboratories (as may be the case due to human use or regulatory considerations), laboratory approaches that are implementable in both austere and well-developed settings may be more desirable than technologically complex methods if the former approach enables data comparisons between locales. As a corollary, laboratory case definitions for epidemiological studies may diverge from those typically used to guide clinical diagnoses.

Finally, longitudinal comparisons will be optimized if the emphasis on harmonized laboratory approaches is matched with the collation of clinical data and the use of proper information systems to catalog both biological samples and their associated clinical datasets.

## Role of vector surveillance

Although this report has focused on surveillance of VBIs in human hosts, entomological vector surveillance also plays a critical role in estimating disease risk and guiding interventions to control transmission. In the absence of a vaccine or prophylactic drug for many VBIs, prevention is reliant on vector control and reduction of infective bites. Optimally, control efforts must be linked with vector and human surveillance data to gauge their effect and estimate disease risk.

A critical distinction between human and vector surveillance is the complexity of vector collection when considering the tremendous diversity of culprit arthropods. In contrast with human surveillance, which generally focuses on disease incidence and/or prevalence in a particular population, unique aspects of vector transmission complicate vector surveillance approaches. For example, for diseases in areas where a specific vector responsible for disease transmission may be unknown or unconfirmed, approaches emphasizing vector identification and transmission competence may need to be prioritized. In other areas where the disease-transmitting potential of arthropod species is well corroborated, targeted characterization of infection rates and vector population densities maximizes the predictive power of field vector surveillance for human VBI cases [33], helping decision makers prioritize vector control efforts and public awareness campaigns [34].

Vector surveillance and control programs face fiscal challenges; they are difficult to sustain because the proportion of infected arthropods may be low and the predictive impact on the acquisition of human disease is difficult to verify. Nonetheless, since surveillance of VBIs in humans is by definition *post factum*, the VBI surveillance approaches must not be neglected since correctly executed vector control measures are uniquely preventive in nature. These factors highlight the need for close coordination between vector and human disease surveillance efforts.

## Conclusion

The studies reviewed in this report highlight the prodigious accomplishments of AFHSC-GEIS’s VBI surveillance program and the potential impact on vector-borne and other communicable diseases of well conceived, relevant and timely surveillance. This review unequivocally demonstrates the benefit provided to host-nation populations through surveillance activities that transcend traditional nation-state boundaries. The capacity of infectious diseases to affect human beings in unforeseen ways, particularly with the advance of VBIs in an ever-shrinking global community, endangers both military and the developing world.

Future efforts of AFHSC-GEIS’s VBI program should center on enhancing the integration of vector, zoonotic and human surveillance activities, continuing to maximize the impact on human diseases of interest to both military and civilian populations, and providing surveillance data that genuinely empowers public health officials. It is essential that the AFHSC-GEIS VBI program remains dedicated to the premise that in the fight against infectious diseases, timely and actionable surveillance data are critical.

## Competing interests

The authors declare that they have no competing interests.
